# Hip Axis Length and Femoral Neck-Shaft Angle as Risk Factors for Proximal Femur Fractures in Octogenarians to Centenarians

**DOI:** 10.3390/jcm13144071

**Published:** 2024-07-12

**Authors:** Daniel Alexandre Gumuchdjian, Manuel Waltenspül, Michael Dietrich, Method Kabelitz

**Affiliations:** Clinic for Orthopaedics, Trauma Surgery and Hand Surgery, Stadtspital Zürich, Tièchestrasse 99, 8037 Zürich, Switzerland; danielalexandre.gumuchdjian@uzh.ch (D.A.G.); manuel.waltenspuel@stadtspital.ch (M.W.); michael.dietrich@stadtspital.ch (M.D.)

**Keywords:** geriatrics, femoral fractures, risk factor, neck-shaft angle, hip axis length

## Abstract

(1) **Background**: The prevention of proximal femoral fractures among people of very advanced age is relevant as they are common and increasing in number. The aim of this study was to determine if the hip axis length (HAL) and the neck-shaft angle (caput–collum–diaphyseal CCD) are risk factors for those fractures among people aged 80 years and over. Consequently, it was additionally analysed if these parameters are associated with a certain fracture type. (2) **Methods**: Anteroposterior radiographs of the pelvis were collected to form three groups (femoral neck fractures (FNFx), trochanteric fractures (TFx) and non-fractured femora (NFx)). Two independent blinded observers separately conducted each measurement of the HAL and CCD. Statistical analysis was performed to determine the association between the measured parameters and type of fracture. (3) **Results**: One hundred and fifty patients (50 per group) were examined, of which the mean age was 92.7 ± 3.5 (range 81–104) years. Both the HAL and CCD of the FNFx group were significantly larger than in the TFx group (*p* = 0.013, 0.003). The CCD was higher in the FNFx than that of the NFx group (*p* = 0.001). No further significant differences of HAL and CCD were observed between the groups. (4) **Conclusions**: For people aged 80 years and over, an increased HAL represented no risk factor for proximal femur fractures, and a large HAL was associated with an increased occurrence of FNFx instead of TFx. A large CCD was associated with an increased risk of suffering a femoral neck fracture, showing evidence of the CCD being a risk factor for the extremely old population.

## 1. Introduction

The prevention of proximal femoral fractures among elderly patients has a high relevance as theses fractures are associated with a high mortality as well as a high dismobility and generate high health care costs [1,2,3,4,5]. Identifying the risk factors for suffering a proximal femoral fracture among very old people would enable to specifically instruct the risk carriers to take preventive measures, e.g., muscle and balance training for reducing the risk of falling [6,7,8,9]. Extensive preventive measures would be justified and sensible if a high risk for fractures is assessed. Hence, finding easy-to-use prognostic risk factors for screening is of importance. Some parameters which are well-established risk factors used in fracture risk assessment tools are, for example, bone density, age, gender and history of previous suffered fractures [10,11,12,13,14,15,16,17,18,19,20,21]. It would be reasonable to examine if the parameters concerning the shape of the femur and the geometric properties of the hip can as well contribute to the fracture risk. Several studies exist which suggest that the hip axis length (HAL) or the femoral neck-shaft angle (caput–collum–diaphyseal angle (CCD)) influence the risk of suffering a proximal femoral fracture [10,22,23,24,25,26,27,28,29]. Due to the demographic shift, the number of very old people with proximal femoral fractures is steadily increasing [1,2,3]. Consequently, it is relevant to find out if these findings can be confirmed on people who are at least 80 years old.

The aim of this study was to determine if the HAL and the CCD are risk factors for proximal femur fractures among people aged 80 years and over. Consequently, it was additionally analysed if these parameters are associated with a certain fracture type.

## 2. Materials and Methods

### 2.1. Data Acquisition

The study was conducted according to the guidelines of the Declaration of Helsinki, and approved by the Ethics Committee of Zürich (BASEC number 2023-01008, 21 July 2023). For the use of the patients’ data, informed consent was given or approval by the ethics commission of Zurich was given.

In this retrospective single-center study, the hospital’s electronical patient database from January 2016 to December 2022 was searched for plain anteroposterior (AP) radiographs of the pelvis.

A total of three groups of patients were formed according to the fracture type, including femoral neck fractures (FNFx), trochanteric fractures (TFx) and non-fractured femora (NFx).

The inclusion criteria for all groups were the presence of adequate plain antero-posterior X-rays of the pelvis fulfilling position standards (knees extended, both legs parallel and 15–20° internally rotated), the patient’s age of at least 80 years at the time the X-ray was taken, the absence of signs for pathological fractures and no documentation of prior femoral fractures bilaterally. For the two fracture groups, additionally a unilateral femur fracture of the corresponding fracture type had to be present to be included. For the NFx group, the additional inclusion criterion was the absence of a femur fracture bilaterally. Patients got excluded if a refusal of participation for this study was documented or their radiographs were of low image quality.

### 2.2. Radiological Measurements

**Figure 1:**
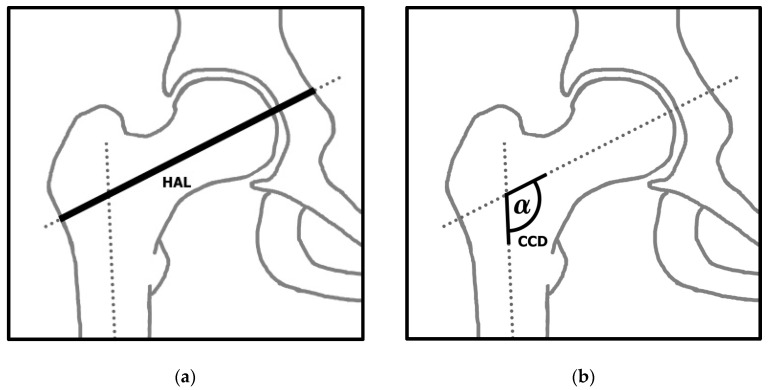
(**a**) Illustration of the hip axis length (HAL); (**b**) illustration of the neck-shaft angle (CCD).

According to Faulkner et al., the HAL is the length along the longitudinal axis of the femoral neck from the base of the trochanter major to the inner pelvic brim (Figure 1a) [24,30]. The CCD is the angle between the longitudinal axis of the femoral neck and the longitudinal axis of the femoral shaft (Figure 1b) [31]. For each radiograph, the HAL and CCD were measured using the clinical software mediCAD (version 6.5, mediCAD Hectec GmbH, Altdorf, Germany).

For both fracture groups, the non-fractured contralateral femur was measured. In the NFx group, the femur, for which an additional axial view radiograph existed, was selected for measuring to ensure consistency.

In order to analyse the inter-observer reliability, two independent blinded observers separately conducted each measurement, whose levels of clinical experience differed (orthopaedic consultant and medical student). To ensure intra-observer reliability, each observer conducted all measurements a second time after a minimum of three weeks. The average of the four measurements was used for data analysis.

### 2.3. Statistical Analysis

By using an a priori power analysis with an alpha of 0.05, it was determined that a sample size of 50 patients per group would ensure a power of over 80%.

The collected data were analysed using descriptive statistics. The Shapiro–Wilk test was applied to assess each group’s measurements of HAL and CCD for normal distribution. In order to assess the equality of variances between the groups, the Levene’s test was used. For the comparison of the continuous variables of the different groups, the independent Student’s *t* test (for normal distribution) and Mann–Whitney U test (for non-normal distribution) were applied. In order to analyse the relationship among the measurements, the Pearson correlation was performed. In order to evaluate the inter- and the intra-observer reliability of the HAL and CCD measurements, this study used the associated 95% confidence interval (95% CI) and the intraclass correlation coefficient (ICC) based on a mean-rating (k = 2), two-way mixed-effects model with consistency definition. Landis and Koch interpreted the strength of agreement using ICC, where an ICC above 0.8 was viewed as almost perfect and a value from 0.6 to 0.8 as substantial [32]. Harris-Hayes et al. interpreted an ICC of 0.96 as excellent [33]. For this study, the software SPSS for Mac (version 23.0, SPSS Inc, Chicago, IL, USA) was used to conduct the statistical analysis, where *p* < 0.05 was selected to determine significance.

## 3. Results

### 3.1. Collected Data and Group Comparison

A total of 150 patients’ radiographs fulfilled the inclusion criteria and passed the exclusion criteria. The overall mean age was 92.7 ± 3.5 (range 81–104) years with a total of 103 women (68.7%) and 47 men (31.3%). For each fracture group (FNFx, TFx and NFx), 50 patients were examined.

The Shapiro–Wilk test showed that the data of the HAL as well as of the CDD were normally distributed within each group. The Levene’s test concluded that an equality of variances existed between all groups for the HAL values as well as for the CCD values.

Table 1 shows the patients’ demographics and results of the radiographic measurements for each group. The largest mean HAL was observed among patients with FNFx, being 4.59 mm longer than in the TFx group, and a significant difference was observed (*p* = 0.013). The HAL of the NFx group showed no significant differences compared to the FNFx group (*p* = 0.065) or to the TFx group (*p* = 0.62). The largest mean CCD was also found in the FNFx group, being 3.38° larger than in the TFx group and 3.76° larger than in the NFx group. A significant difference of the CCD was observed between patients with FNFx and NFx (*p* = 0.001). The CCD of the FNFx group was also significantly larger than in the TFx group (*p* = 0.003). No significant difference of the CCD was found between patients with TFx and NFx (*p* = 0.739) as these groups showed similar statistical results. The results of the statistical analysis are presented in Table 2.

**Figure 2:**
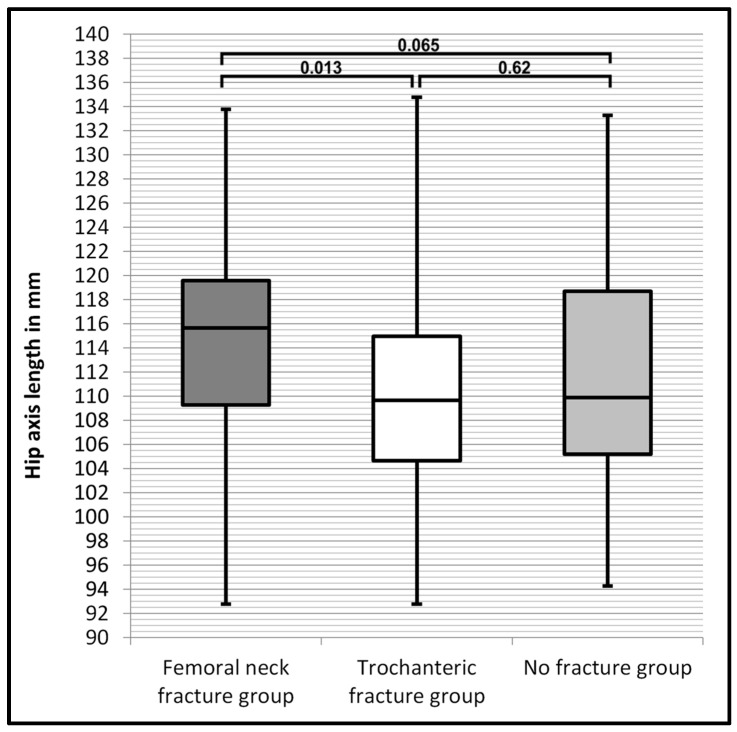
Box plot diagram comparing values of the HAL of each group and showing the corresponding *p*-values between the groups.

**Figure 3:**
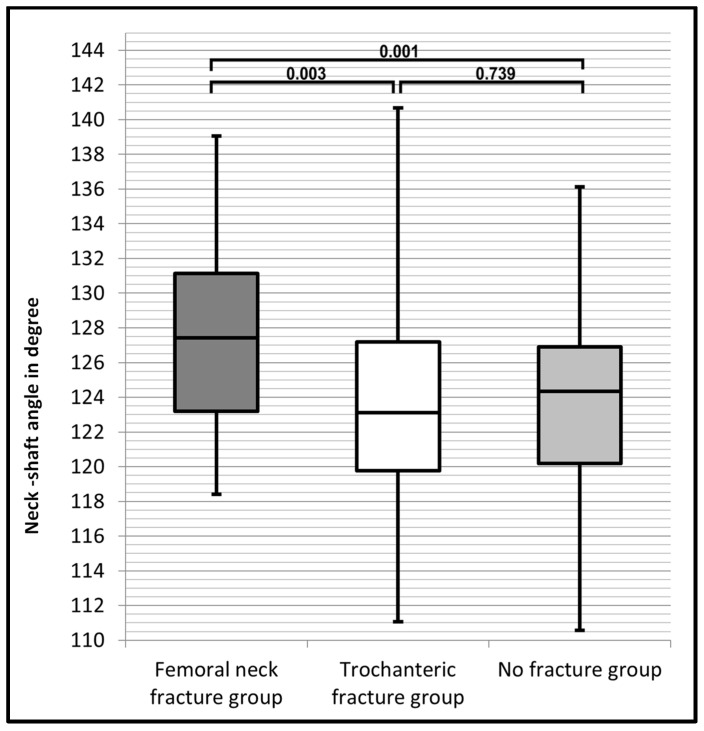
Box plot diagram comparing values of the CCD of each group and showing the corresponding *p*-values between the groups.

The distribution of the HAL and the CCD in the different groups is displayed in Figure 2 and Figure 3, visualising the differences of range, interquartile range and median.

Figure 2 shows that the ranges of the HAL were similar in all groups. In the FNFx group (dark grey box), the lower and upper limit of the interquartile range of the HAL were both about 5 mm larger than in the TFx group (white box). The NFx group (light grey box) had the widest spread interquartile range. The FNFx group had the largest median HAL. The median HAL of the TFx group and the NFx group was similar.

Figure 3 shows that the CCD of the FNFx group (dark grey box) had the largest lower and upper limit of its interquartile range. For their CCD measurements, the TFx group (white box) and the NFx group (light grey box) had a similar interquartile range. The CCD of the FNFx group had the narrowest spread of the range and the largest median.

### 3.2. Inter- and Intra-Rater Reliability

The inter-rater reliability for the HAL measurements was excellent, showing ICCs of almost perfect strength of agreement for all groups (FNFx 0.991, TFx 0.993, NFx 0.997; *p* < 0.001). For the CCD measurements, the ICCs were also excellent (FNFx 0.936, TFx 0.944, NFx 0.974; *p* < 0.001). The intra-rater reliability was equally excellent, showing a very high correlation of the measurements of the HAL (FNFx 0.996, TFx 0.994, NFx 0.996; *p* < 0.001) and of the CCD (FNFx 0.963, TFx 0.961, NFx 0.969; *p* < 0.001).

## 4. Discussion

In this study, which for the first time presents data for patients of such an advanced average age, the HAL in the FNFx group was significantly larger than in the TFx group, and the NFx group showed no significantly different HAL compared to both fracture groups. Therefore, having a large HAL is associated with an increased risk to suffer FNFx instead of TFx. Hence, this analysis shows that among very old patients, the HAL can serve as a potential prognostic factor to predict which type of proximal femoral fracture is likelier to occur. Additionally, the results have demonstrated that the CCD of the FNFx group was significantly larger than in the TFx group and the NFx group. This indicates that among people aged 80 years and over, a large CCD can be associated with FNFx and increases the risk of suffering a proximal femur fracture.

In the literature, data for geriatric patients in their eighth to tenth decade of life concerning this issue are sparsely covered, since most studies examined younger patients. Some studies conducted their examination without differentiating between fracture types and therefore their results could not be compared and discussed in this study [10,23,27,28,29].

Several previous studies, which compared the HAL of the FNFx group with the NFx group, examined only women, finding a significantly larger HAL in the FNFx group [25,30,34,35]. In contrast, other studies, which examined both sexes, found no significant difference [36,37]. Our study, which also included both sexes, has neither found a significantly different HAL between the FNFx and the NFX group, indicating that sex is a decisive factor for the outcome.

Numerous studies comparing the HAL of the TFx with the NFx group found no significant difference [25,34,35,37]. In contrast, few studies found a significantly larger HAL among TFx patients [30,36]. Our outcome concords with the findings of the majority of these previous studies.

Few studies conducted a comparison of the HAL between patients with FNFx and with TFx, finding no significant difference, and the average age of their participants was between 76.3 and 78.6 years [37,38]. This study, on the contrary, which included participants with a mean age of 92.7 years, found a significantly larger HAL in the FNFx group, showing that among very old people, the results differ significantly.

Several previous studies examining the CCD of patients with FNFx and NFx reported a significantly larger CCD in the FNFx group [25,35,36]. Contrarily, Ito et al. could not detect a significant difference [34]. The findings of this study concord with the results of the majority of these previous studies.

Numerous previous studies, which examined the CCD of the TFx group and NFx group, found a significantly larger CCD in the TFx group [34,36,37]. In contrast, Gnudi et al. observed no significant difference [25]. Our outcome matches the findings of the majority of these previous studies.

Few studies compared the CCD between patients with FNFx and with TFx, observing no significant difference [37,38]. In contrast, we did observe between the FNFx and the TFx group a significantly larger CCD in the FNFx group. This divergence might be again explained by the much higher average age of our participants.

The strength of this study is its very high precision of measurements, which is proven by the excellent correlation in the assessment of the inter- and intra-rater reliability. To ensure accurate results, a specific age group of geriatric patients was examined, enabling a more comparable general health status of the participants.

As this is a retrospective study, the obvious limitation of its retrospective bias has to be taken into consideration. In this study for the fracture groups, the contralateral femur was measured as a reference under the assumption that both femora have an identical HAL and CCD. Studies have shown that although a strong symmetry between both femora exists, minor differences can occur [39,40,41]. Additionally, minor radiographic distortions are possible due to small deviations from position standards. Therefore, minor divergences between the used values and the real values of the HAL and the CCD could not be excluded. Also, the proportion of male participants was not identical in each group. This might have affected the results as some studies stated that the HAL is influenced by sex; on the other hand, for the CCD, the studies’ results were contradictory, either stating or rejecting the relevance of sex [42,43,44,45,46,47].

According to this study, the HAL is not an adequate screening parameter to assess the fracture risk among very old people. This study shows evidence of the CCD being a risk factor for suffering a femoral neck fracture for people 80 years and older. Therefore, it would be reasonable in geriatrics to advise very old patients with a large CDD to take preventive measures to reduce the risk of falling.

If the CCD were added as a further parameter in multiple factor fracture risk assessment tools for people aged 80 years and over, the risk prediction would probably become more accurate. Examining this assumption in future studies would be valuable.

This study provides an important contribution to the literature as it is the first study about this subject for this age group, which is growing in number.

## 5. Conclusions

For people aged 80 years and over. an increased HAL represents no risk factor for proximal femur fractures, and a large HAL is associated with an increased occurrence of FNFx instead of TFx. A larger CCD is associated with an increased risk of suffering from FNFx, showing evidence of the CCD being a risk factor for people of very advanced age.

## Figures and Tables

**Table 1:** Patients’ demographics and measuring results for each group.

	FNFx Group	TFx Group	NFx Group
Number of participants	50	50	50
Age (years) ^1^	93.8 ± 3.2	92.9 ± 3.7	91.3 ± 3.2
Sex (female/male)	30/20	38/12	35/15
HAL (mm) ^1^	115.24 ± 9.50	110.65 ± 8.54	111.58 ± 10.05
HAL range (mm)	92.8–133.8	92.8–134.8	94.3–133.3
CCD (°) ^1^	127.41 ± 5.26	124.03 ± 5.98	123.65 ± 5.43
CCD range (°)	118.4–139.1	111.1–140.7	110.6–136.1

^1^ data shown as mean ± standard deviation. FNFx: femoral neck fractures; TFx: trochanteric fractures; NFx: non-fractured femora; HAL: hip axis length; CCD: neck-shaft angle.

**Table 2:** Results of statistical analysis (*p*—values).

	FNFx vs. TFx	FNFx vs. NFx	TFx vs. NFx
HAL ^1^	0.013	0.065	0.62
CCD ^1^	0.003	0.001	0.739

^1^ *p* < 0.05 equals a statistical significance. FNFx: femoral neck fractures; TFx: trochanteric fractures; NFx: non-fractured femora; HAL: hip axis length; CCD: neck-shaft angle.

## Data Availability

Access to the data can be granted by the corresponding author upon request.
